# Crystal structure of bis­(4-meth­oxy­pyridine-κ*N*)(*meso*-5,10,15,20-tetra­phenyl­porphyrinato-κ^4^ *N*,*N*′,*N*′′,*N*′′′)iron(III) perchlorate

**DOI:** 10.1107/S2056989019006194

**Published:** 2019-05-10

**Authors:** Morten K. Peters, Christian Näther, Rainer Herges

**Affiliations:** a Otto-Diels-Institut für Organische Chemie, Christian-Albrechts-Universität Kiel, Otto-Hahn-Platz 4, D-24098 Kiel, Germany; b Institut für Anorganische Chemie, Christian-Albrechts-Universität Kiel, Max-Eyth Str. 2, D-24118 Kiel, Germany

**Keywords:** crystal structure, hydrogen bonding, spin crossover, P450, iron(III) porphyrin

## Abstract

In the crystal structure of the title compound, [Fe(C_44_H_28_N_4_)(C_6_H_7_NO)_2_]ClO_4_, the Fe^III^ ions are coordinated in an octa­hedral fashion by four N atoms of the porphyrin moiety and two N atoms of two 4-meth­oxy­pyridine ligands into discrete complexes that are located on inversion centers. Charge-balance is achieved by perchlorate anions that are disordered around twofold rotation axes. In the crystal structure, the discrete cationic complexes and the perchlorate anions are arranged into layers with weak C—H⋯O inter­actions between the cations and the anions. The porphyrin moieties of neighboring layers show a herringbone-like arrangement.

## Abstract 2

In the crystal structure of the title compound, the Fe^III^ ions are octa­hedrally coordinated by four N atoms of a porphyrin moiety as well as two 4-meth­oxy­pyridine ligands into discrete complexes that are charge-balanced by perchlorate anions.

## Chemical context   

Porphyrins are of great inter­est for a number of different applications in medicine and nature (Peters & Herges, 2018 ▸; Peters *et al.*, 2018 ▸; Shankar *et al.*, 2018 ▸; Dommaschk *et al.*, 2015 ▸). For example, metal porphyrins show spin crossover (SCO), which is the key step in a number of enzymatic reactions, *e.g*. catalysts in selective CH activation (cytochrome P450) (Konishi *et al.*, 1992 ▸; Momenteau *et al.*, 1983 ▸), hydrogen peroxide decomposition (catalases) (Maté *et al.*, 2001 ▸) and a number of other biologically important processes (Collman *et al.*, 1995 ▸; Gunter *et al.*, 1994 ▸; Morgan & Dolphin, 1987 ▸). The spin state and electronic configuration of ferrous porphyrins are dependent on temperature, pressure, light or axial ligands.
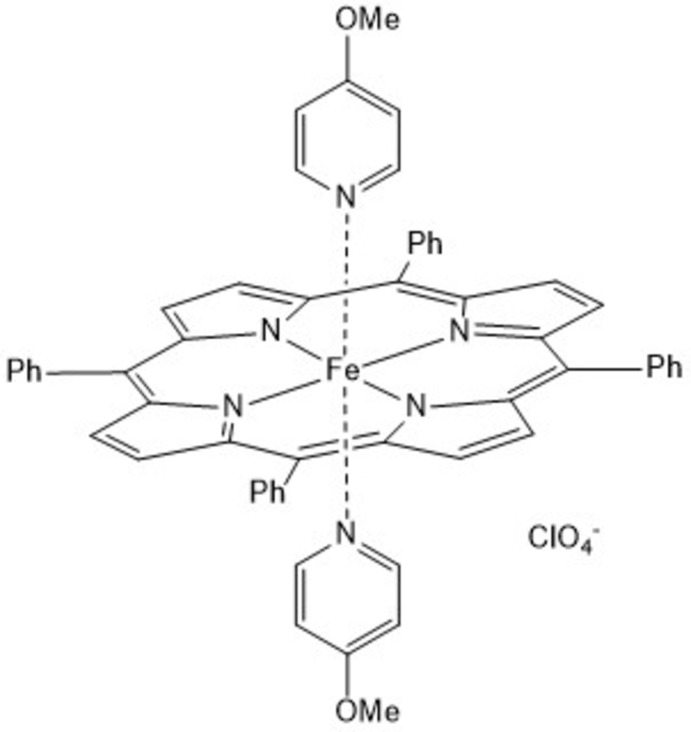


Iron(III) porphyrins can exist in high-spin (*S* = 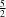), inter­mediate-spin (*S* = 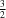), admixed-spin (*S* = 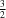, 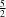) and low-spin (*S* = 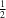) states of iron (Scheidt, 2000 ▸; Ikezaki *et al.*, 2009 ▸; Nakamura, 2006 ▸; Shankar *et al.*, 2018 ▸). Most of the anionic ligands such as chloride, hydroxide and azide lead to the formation of complexes in the high-spin state, whereas weak ligands like ClO_4_^−^ and SbF_6_^−^ usually give the complexes in an admixed-spin state (Scheidt, 2000 ▸). However, six-coordinate complexes with strong axial ligands tend to be in the low-spin state (Scheidt, 2000 ▸). In our ongoing investigations on SCO compounds based on iron porphyrins, we became inter­ested in the complex bis­(4-meth­oxy­pyridine-κ*N*)(*meso*-5,10,15,20-tetra­phenyl­porphyrinato-κ^4^ *N*,*N*′,*N*′′,*N*′′′iron(III) perchlorate, which was synthesized and characterized by high-resolution mass spectroscopy (Shankar *et al.*, 2018 ▸). Preliminary investigations indicate that the complex is in the low-spin state but unfortunately no single crystals were obtained. In the course of subsequent investigations, we we able to obtain crystals by the layering technique starting from the Fe^III^ tetra­phenyl­porphyrin perchlorate complexes and using 4-meth­oxy­pyridine dissolved in di­chloro­methane as the lower and *n*-heptane as the upper layer. These crystals were identified by single crystal X-ray diffraction, which confirmed that crystals of the title compound were obtained.

## Structural commentary   

**Figure 1:**
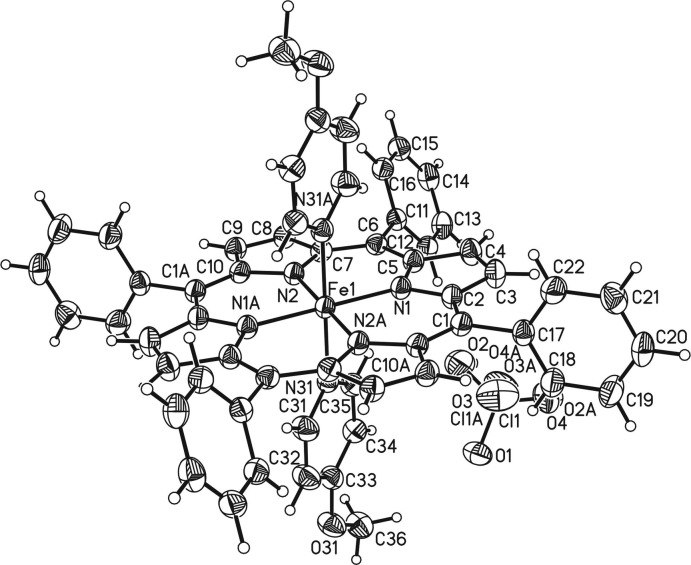
Mol­ecular structure of the title compound with displacement ellipsoids drawn at the 50% probability level. Atoms with the suffix A are generated by the symmetry operation (1 − *x*, 1 − *y*, 1 − *z*).

**Figure 2:**
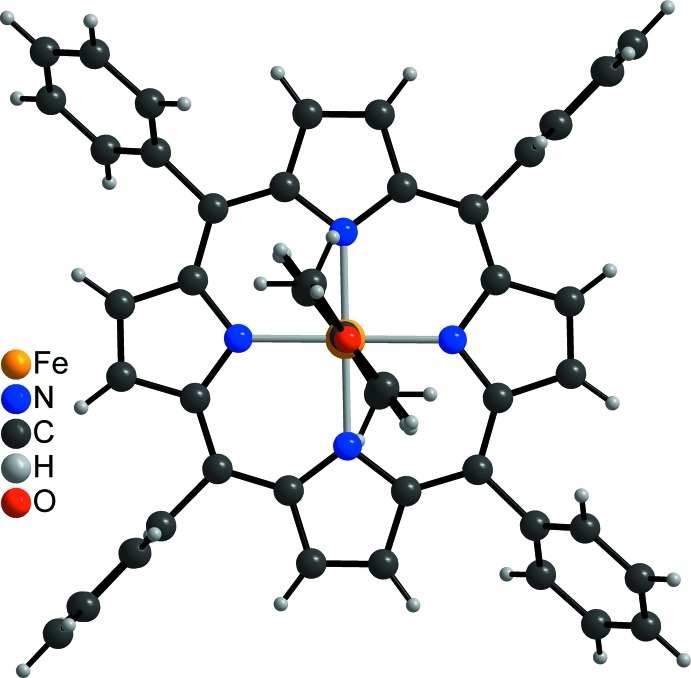
Mol­ecular structure of the title compound viewed onto the porphyrin plane.

The crystal structure of the title compound consists of discrete complexes which lie on inversion centers. The Fe^III^ ions are sixfold coordinated by four N atoms of the porphyrin moiety and two N atoms of two 4-meth­oxy­pyridine ligands in an octa­hedral coordination environment (Fig. 1 ▸). The Fe—N bond lengths to the porphyrin atoms of 1.9989 (13) Å and to the pyridine N atoms of 2.0002 (13) Å are nearly identical and the iron cations are located exactly in the plane of the coordinating porphyrin N atoms (Table 1 ▸). The Fe—N bond lengths to the two axial 4-meth­oxy­pyridine ligands at 2.0 Å are typical for low-spin complexes (Geiger *et al.*, 1985 ▸; Scheidt & Geiger, 1979 ▸), whereas high-spin complexes have a significant longer bond length of about 2.2 Å (Geiger *et al.*, 1984 ▸, 1985 ▸; Geiger & Scheidt, 1984 ▸). The N—Fe—N bond angles within the equatorial porphyrin plane range between 88.56 (5) and 91.44 (5)°, whereas that to the axial ligands are 180° because of symmetry, which proves that the octa­hedra are slightly distorted (Table 1 ▸). The six-membered ring planes of the two coordinating 4-meth­oxy­pyridine ligands are eclipsed and rotated relative to the Fe—N bonds of the Fe^III^-porphyrin moiety (Fig. 2 ▸). Two of the four phenyl rings are nearly perpendicular to the porphyrin ring planes with a dihedral angle of 87.82 (5)°, whereas the other two rings are rotated out of this plane by 63.64 (5)°. The positive charge of the Fe^III^-porphyrin moiety is compensated by one perchlorate anion that is disordered around a twofold rotation axis.

## Supra­molecular features   

**Figure 3:**
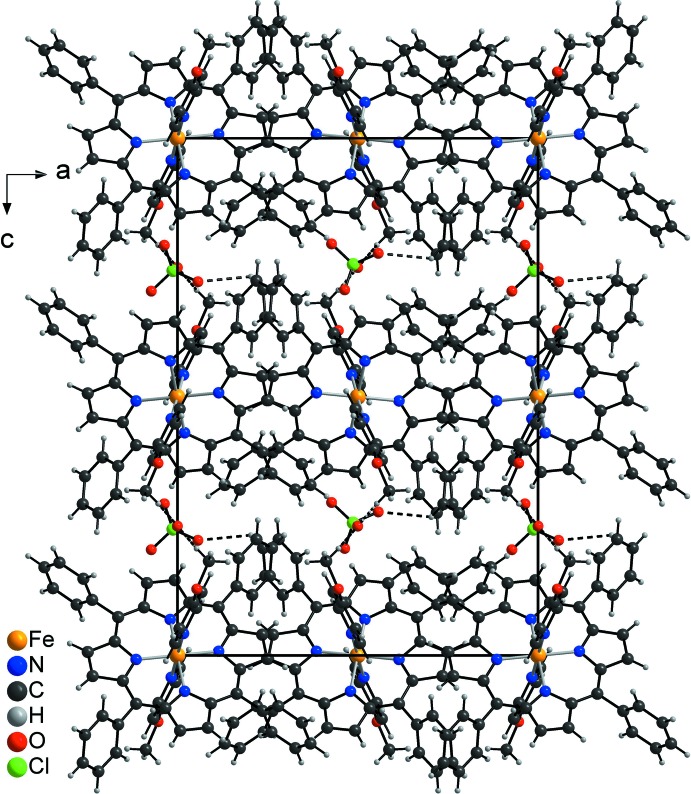
Crystal packing of the title compound viewed along the *b* axis. Inter­molecular C—H⋯O contacts are shown as dashed lines.

**Figure 4:**
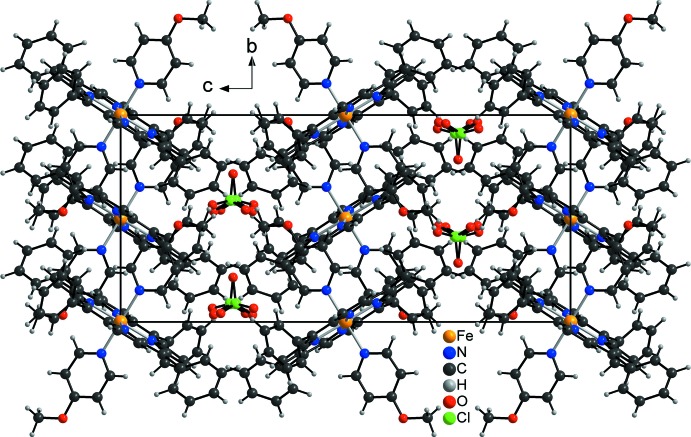
Crystal packing of the title compound viewed along the *a* axis.

In the crystal structure, the Fe-porphyrin cations and the perchlorate anions are each arranged in layers that are located parallel to the *ab* plane (Fig. 3 ▸). These layers are connected to the perchlorate anions by weak C—H⋯O contacts (Table 2 ▸). For one of these contacts, the C—H⋯O angle is close to linearity, indicating weak inter­molecular hydrogen bonding (Fig. 3 ▸ and Table 2 ▸). The porphyrin units of neighboring layers exhibit a herringbone-like arrangement (Fig. 4 ▸).

## Database survey   

According to a search in the Cambridge Structural Database (CSD Version 5.4, update of February 2019; Groom *et al.*, 2016 ▸), 1009 structures of ferrous porphyrins have been reported. However, ferrous porphyrins with axial 4-meth­oxy­pyridine ligands are unknown although ferrous porphyrins with perchlorate as counter-ion and other pyridines as axial ligands have been published, for instance the sterically congested porphyrin (2,3,7,8,12,13,17,18-octa­methyl-5,10,15,20-tetra­phenyl­porph­yrinato)iron(III) perchlorate which has two pyridine mol­ecules as axial ligands (Ohgo *et al.*, 2002 ▸, 2004 ▸). Other iron(III) porphyrin perchlorates are known with 3-chloro­pyridine (Scheidt & Geiger, 1979 ▸), 4-cyano­pyridine (Safo *et al.*, 1994 ▸), 3,5-di­chloro­pyridine (Scheidt *et al.*, 1989 ▸) and 4-cyano­pyridine ligands (Yatsunyk & Walker 2004 ▸; Safo *et al.* 1994 ▸; Safo *et al.* 1992 ▸).

## Synthesis and crystallization   

Fe^III^ tetra­phenyl­porphyrin perchlorate (FeTPPClO_4_) was synthesized as previously reported (Shankar *et al.*, 2018 ▸). The layering technique was used for crystallization. The lower layer was di­chloro­methane with 50 µL 4-meth­oxy­pyridine and *n*-heptane was used for the upper anti­solvent.

## Refinement   

Crystal data, data collection and structure refinement details are summarized in Table 3 ▸. The C—H hydrogen atoms were positioned with idealized geometries (C—H = 0.95–0.98 Å; methyl H atoms allowed to rotate but not to tip) and were refined isotropically using a riding model with *U*_iso_(H) = 1.2*U*_eq_(C) or 1.5*U*_eq_(C-meth­yl). The perchlorate anion is disordered around a twofold rotation axis that passes through O1 and thus, disordered because of symmetry.

## Supplementary Material

Crystal structure: contains datablock(s) I. DOI: 10.1107/S2056989019006194/lh5899sup1.cif

Structure factors: contains datablock(s) I. DOI: 10.1107/S2056989019006194/lh5899Isup2.hkl

CCDC reference: 1913651

Additional supporting information:  crystallographic information; 3D view; checkCIF report

## Figures and Tables

**Table 1:** Selected geometric parameters (Å, °)

Fe1—N1	1.9989 (13)	Fe1—N2	2.0003 (13)
Fe1—N2^i^	2.0002 (13)	Fe1—N31	2.0177 (14)
			
N1^i^—Fe1—N1	180.00 (4)	N1—Fe1—N31	91.13 (5)
N1—Fe1—N2^i^	91.44 (5)	N2—Fe1—N31	89.64 (6)
N1—Fe1—N2	88.56 (5)	N2—Fe1—N31^i^	90.36 (6)
N2^i^—Fe1—N2	180.00 (8)	N31—Fe1—N31^i^	180.0
N1^i^—Fe1—N31	88.87 (6)		

Symmetry code: (i) 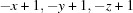.

**Table 2:** Hydrogen-bond geometry (Å, °)

*D*—H⋯*A*	*D*—H	H⋯*A*	*D*⋯*A*	*D*—H⋯*A*
C35—H35⋯O4^ii^	0.95	2.55	3.103 (8)	117
C36—H36*C*⋯O1	0.98	2.64	3.551 (3)	154

Symmetry code: (ii) 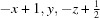.

**Table 3:** Experimental details

Crystal data	
Chemical formula	[Fe(C_44_H_28_N_4_)(C_6_H_7_NO)_2_]ClO_4_
*M*_r_	986.25
Crystal system, space group	Orthorhombic, *P* *b* *c* *n*
Temperature (K)	170
*a*, *b*, *c* (Å)	16.9772 (4), 11.1879 (2), 24.3484 (6)
*V* (Å^3^)	4624.72 (18)
*Z*	4
Radiation type	Mo *K*α
μ (mm^−1^)	0.45
Crystal size (mm)	0.12 × 0.10 × 0.09

Data collection	
Diffractometer	Stoe IPDS2
No. of measured, independent and observed [*I* > 2σ(*I*)] reflections	36542, 5029, 4454
*R*_int_	0.033
(sin θ/λ)_max_ (Å^−1^)	0.639

Refinement	
*R*[*F* ^2^ > 2σ(*F* ^2^)], *wR*(*F* ^2^), *S*	0.040, 0.099, 1.07
No. of reflections	5029
No. of parameters	337
H-atom treatment	H-atom parameters constrained
Δρ_max_, Δρ_min_ (e Å^−3^)	0.28, −0.48

Computer programs: *X-AREA* (Stoe & Cie, 2008 ▸), *SHELXS97* (Sheldrick, 2008 ▸), *SHELXL2014* (Sheldrick, 2015 ▸), *XP* (Sheldrick, 2008 ▸), *DIAMOND* (Brandenburg, 2014 ▸) and *publCIF* (Westrip, 2010 ▸).

## supplementary crystallographic information

### Crystal data

**Table:**

[Fe(C_44_H_28_N_4_)(C_6_H_7_NO)_2_]ClO_4_	*D*_x_ = 1.416 Mg m^−^^3^
*M**_r_* = 986.25	Mo *K*α radiation, λ = 0.71073 Å
Orthorhombic, *P**b**c**n*	Cell parameters from 36542 reflections
*a* = 16.9772 (4) Å	θ = 1.7–27.0°
*b* = 11.1879 (2) Å	µ = 0.45 mm^−^^1^
*c* = 24.3484 (6) Å	*T* = 170 K
*V* = 4624.72 (18) Å^3^	Block, colorless
*Z* = 4	0.12 × 0.10 × 0.09 mm
*F*(000) = 2044	

### Data collection

**Table:**

STOE IPDS-2 diffractometer	*R*_int_ = 0.033
ω scans	θ_max_ = 27.0°, θ_min_ = 1.7°
36542 measured reflections	*h* = −21→21
5029 independent reflections	*k* = −13→14
4454 reflections with *I* > 2σ(*I*)	*l* = −29→31

### Refinement

**Table:**

Refinement on *F*^2^	0 restraints
Least-squares matrix: full	Hydrogen site location: inferred from neighbouring sites
*R*[*F*^2^ > 2σ(*F*^2^)] = 0.040	H-atom parameters constrained
*wR*(*F*^2^) = 0.099	*w* = 1/[σ^2^(*F*_o_^2^) + (0.0472*P*)^2^ + 2.1988*P*] where *P* = (*F*_o_^2^ + 2*F*_c_^2^)/3
*S* = 1.07	(Δ/σ)_max_ < 0.001
5029 reflections	Δρ_max_ = 0.28 e Å^−^^3^
337 parameters	Δρ_min_ = −0.48 e Å^−^^3^

### Special details

**Table:**

Geometry. All esds (except the esd in the dihedral angle between two l.s. planes) are estimated using the full covariance matrix. The cell esds are taken into account individually in the estimation of esds in distances, angles and torsion angles; correlations between esds in cell parameters are only used when they are defined by crystal symmetry. An approximate (isotropic) treatment of cell esds is used for estimating esds involving l.s. planes.

### Fractional atomic coordinates and isotropic or equivalent isotropic displacement parameters (Å^2^)

**Table:**

	*x*	*y*	*z*	*U*_iso_*/*U*_eq_	Occ. (<1)
Fe1	0.5000	0.5000	0.5000	0.02670 (10)	
N1	0.51998 (8)	0.59036 (13)	0.43056 (5)	0.0287 (3)	
N2	0.38550 (8)	0.53845 (13)	0.49210 (5)	0.0285 (3)	
C1	0.66431 (9)	0.56717 (15)	0.42585 (6)	0.0303 (3)	
C2	0.59265 (9)	0.61091 (16)	0.40705 (7)	0.0312 (3)	
C3	0.58436 (10)	0.68660 (18)	0.36008 (7)	0.0386 (4)	
H3	0.6257	0.7136	0.3369	0.046*	
C4	0.50747 (10)	0.71259 (17)	0.35457 (7)	0.0374 (4)	
H4	0.4844	0.7618	0.3271	0.045*	
C5	0.46682 (9)	0.65166 (15)	0.39806 (6)	0.0301 (3)	
C6	0.38565 (9)	0.65732 (15)	0.40647 (6)	0.0294 (3)	
C7	0.34876 (9)	0.60222 (15)	0.45098 (6)	0.0295 (3)	
C8	0.26493 (9)	0.60202 (16)	0.45999 (7)	0.0322 (3)	
H8	0.2263	0.6386	0.4373	0.039*	
C9	0.25149 (10)	0.54030 (17)	0.50662 (7)	0.0332 (3)	
H9	0.2016	0.5257	0.5230	0.040*	
C10	0.32645 (9)	0.50074 (15)	0.52689 (7)	0.0293 (3)	
C11	0.33579 (9)	0.72443 (15)	0.36631 (6)	0.0294 (3)	
C12	0.33009 (10)	0.68687 (16)	0.31184 (7)	0.0341 (4)	
H12	0.3600	0.6203	0.2996	0.041*	
C13	0.28082 (10)	0.74660 (19)	0.27552 (7)	0.0390 (4)	
H13	0.2768	0.7199	0.2386	0.047*	
C14	0.23762 (10)	0.84445 (19)	0.29253 (8)	0.0415 (4)	
H14	0.2040	0.8849	0.2675	0.050*	
C15	0.24377 (10)	0.88294 (17)	0.34625 (8)	0.0395 (4)	
H15	0.2145	0.9506	0.3581	0.047*	
C16	0.29235 (10)	0.82350 (16)	0.38300 (7)	0.0341 (4)	
H16	0.2960	0.8506	0.4199	0.041*	
C17	0.73642 (9)	0.59461 (16)	0.39261 (7)	0.0312 (3)	
C18	0.76060 (12)	0.51717 (19)	0.35181 (8)	0.0437 (4)	
H18	0.7316	0.4461	0.3450	0.052*	
C19	0.82706 (12)	0.5424 (2)	0.32064 (9)	0.0500 (5)	
H19	0.8438	0.4878	0.2931	0.060*	
C20	0.86880 (10)	0.6461 (2)	0.32953 (8)	0.0434 (4)	
H20	0.9137	0.6640	0.3077	0.052*	
C21	0.84516 (11)	0.72356 (19)	0.37005 (9)	0.0460 (5)	
H21	0.8740	0.7950	0.3764	0.055*	
C22	0.77939 (11)	0.69788 (18)	0.40173 (8)	0.0423 (4)	
H22	0.7637	0.7516	0.4299	0.051*	
N31	0.48297 (8)	0.34725 (13)	0.45758 (6)	0.0299 (3)	
C31	0.50607 (12)	0.23963 (17)	0.47644 (8)	0.0420 (4)	
H31	0.5320	0.2360	0.5110	0.050*	
C32	0.49415 (13)	0.13525 (18)	0.44838 (8)	0.0457 (5)	
H32	0.5116	0.0614	0.4633	0.055*	
C33	0.45604 (11)	0.13819 (16)	0.39757 (7)	0.0363 (4)	
C34	0.43316 (10)	0.24801 (16)	0.37747 (7)	0.0351 (4)	
H34	0.4077	0.2541	0.3429	0.042*	
C35	0.44775 (10)	0.34847 (16)	0.40822 (7)	0.0342 (4)	
H35	0.4319	0.4235	0.3937	0.041*	
O31	0.44631 (9)	0.03299 (12)	0.37175 (6)	0.0467 (3)	
C36	0.41268 (13)	0.03669 (19)	0.31772 (8)	0.0461 (5)	
H36A	0.3605	0.0738	0.3194	0.069*	
H36B	0.4078	−0.0448	0.3034	0.069*	
H36C	0.4468	0.0836	0.2934	0.069*	
Cl1	0.51249 (9)	0.41187 (7)	0.25605 (9)	0.0354 (3)	0.5
O1	0.5000	0.28459 (18)	0.2500	0.0480 (5)	
O2	0.4431 (3)	0.4585 (8)	0.2775 (3)	0.084 (3)	0.5
O3	0.5724 (2)	0.4344 (3)	0.29567 (16)	0.0722 (10)	0.5
O4	0.5362 (5)	0.4650 (9)	0.2069 (3)	0.095 (3)	0.5

### Atomic displacement parameters (Å^2^)

**Table:**

	*U*^11^	*U*^22^	*U*^33^	*U*^12^	*U*^13^	*U*^23^
Fe1	0.02464 (16)	0.02978 (17)	0.02567 (16)	0.00148 (12)	0.00190 (11)	0.00273 (12)
N1	0.0254 (6)	0.0324 (7)	0.0282 (6)	0.0014 (5)	0.0020 (5)	0.0033 (5)
N2	0.0259 (6)	0.0325 (7)	0.0272 (6)	0.0018 (5)	0.0017 (5)	0.0032 (5)
C1	0.0285 (7)	0.0334 (9)	0.0292 (8)	0.0003 (6)	0.0032 (6)	0.0012 (6)
C2	0.0294 (8)	0.0347 (9)	0.0296 (8)	0.0003 (6)	0.0044 (6)	0.0042 (7)
C3	0.0323 (8)	0.0479 (11)	0.0357 (9)	0.0010 (7)	0.0057 (7)	0.0118 (8)
C4	0.0324 (8)	0.0431 (10)	0.0367 (9)	0.0022 (7)	0.0018 (7)	0.0116 (8)
C5	0.0296 (8)	0.0327 (9)	0.0281 (7)	0.0023 (6)	0.0002 (6)	0.0047 (6)
C6	0.0294 (7)	0.0302 (8)	0.0288 (7)	0.0025 (6)	0.0003 (6)	0.0005 (6)
C7	0.0276 (7)	0.0322 (8)	0.0286 (8)	0.0023 (6)	−0.0011 (6)	0.0005 (6)
C8	0.0269 (8)	0.0377 (9)	0.0321 (8)	0.0034 (7)	−0.0008 (6)	0.0029 (7)
C9	0.0264 (7)	0.0382 (9)	0.0349 (8)	0.0020 (7)	0.0022 (6)	0.0019 (7)
C10	0.0263 (7)	0.0322 (8)	0.0293 (8)	0.0006 (6)	0.0022 (6)	0.0003 (6)
C11	0.0268 (7)	0.0315 (8)	0.0300 (8)	0.0003 (6)	0.0004 (6)	0.0044 (6)
C12	0.0339 (8)	0.0364 (9)	0.0321 (8)	0.0007 (7)	0.0021 (6)	0.0016 (7)
C13	0.0348 (8)	0.0528 (11)	0.0294 (8)	−0.0045 (8)	−0.0030 (7)	0.0058 (8)
C14	0.0305 (8)	0.0516 (11)	0.0424 (9)	0.0022 (8)	−0.0026 (7)	0.0168 (9)
C15	0.0325 (8)	0.0375 (10)	0.0486 (10)	0.0074 (7)	0.0047 (8)	0.0096 (8)
C16	0.0334 (8)	0.0351 (9)	0.0339 (8)	0.0019 (7)	0.0024 (7)	0.0022 (7)
C17	0.0262 (7)	0.0366 (9)	0.0309 (8)	0.0020 (6)	0.0014 (6)	0.0063 (7)
C18	0.0439 (10)	0.0459 (11)	0.0414 (10)	−0.0082 (8)	0.0113 (8)	−0.0035 (8)
C19	0.0459 (11)	0.0614 (13)	0.0427 (10)	−0.0035 (10)	0.0156 (8)	−0.0055 (10)
C20	0.0288 (8)	0.0622 (13)	0.0394 (9)	−0.0021 (8)	0.0052 (7)	0.0110 (9)
C21	0.0332 (9)	0.0473 (11)	0.0574 (12)	−0.0083 (8)	0.0014 (8)	0.0054 (9)
C22	0.0356 (9)	0.0424 (10)	0.0489 (10)	−0.0017 (8)	0.0081 (8)	−0.0037 (8)
N31	0.0284 (6)	0.0325 (7)	0.0289 (6)	0.0019 (5)	0.0009 (5)	0.0019 (6)
C31	0.0550 (11)	0.0366 (10)	0.0342 (9)	0.0036 (8)	−0.0073 (8)	0.0035 (7)
C32	0.0652 (13)	0.0338 (10)	0.0381 (10)	0.0025 (9)	−0.0075 (9)	0.0052 (8)
C33	0.0404 (9)	0.0330 (9)	0.0355 (9)	−0.0041 (7)	0.0010 (7)	0.0001 (7)
C34	0.0341 (8)	0.0388 (9)	0.0324 (8)	0.0020 (7)	−0.0034 (7)	0.0005 (7)
C35	0.0352 (8)	0.0349 (9)	0.0325 (8)	0.0040 (7)	−0.0017 (6)	0.0021 (7)
O31	0.0652 (9)	0.0333 (7)	0.0416 (7)	−0.0056 (6)	−0.0088 (6)	−0.0011 (6)
C36	0.0558 (12)	0.0411 (10)	0.0413 (10)	−0.0094 (9)	−0.0083 (9)	−0.0030 (8)
Cl1	0.0332 (11)	0.0327 (3)	0.0404 (10)	0.0001 (4)	0.0032 (6)	−0.0014 (4)
O1	0.0637 (13)	0.0331 (10)	0.0472 (11)	0.000	−0.0063 (9)	0.000
O2	0.044 (2)	0.053 (3)	0.154 (8)	0.0169 (19)	0.050 (3)	−0.008 (4)
O3	0.066 (2)	0.067 (2)	0.083 (2)	−0.0006 (18)	−0.0318 (19)	−0.0234 (19)
O4	0.184 (9)	0.051 (3)	0.050 (3)	−0.006 (5)	0.049 (4)	0.006 (2)

### Geometric parameters (Å, º)

**Table:**

Fe1—N1^i^	1.9988 (13)	C17—C22	1.384 (3)
Fe1—N1	1.9989 (13)	C18—C19	1.389 (3)
Fe1—N2^i^	2.0002 (13)	C18—H18	0.9500
Fe1—N2	2.0003 (13)	C19—C20	1.377 (3)
Fe1—N31	2.0177 (14)	C19—H19	0.9500
Fe1—N31^i^	2.0177 (14)	C20—C21	1.373 (3)
N1—C2	1.379 (2)	C20—H20	0.9500
N1—C5	1.382 (2)	C21—C22	1.387 (3)
N2—C10	1.379 (2)	C21—H21	0.9500
N2—C7	1.379 (2)	C22—H22	0.9500
C1—C10^i^	1.388 (2)	N31—C35	1.342 (2)
C1—C2	1.389 (2)	N31—C31	1.347 (2)
C1—C17	1.499 (2)	C31—C32	1.368 (3)
C2—C3	1.430 (2)	C31—H31	0.9500
C3—C4	1.344 (2)	C32—C33	1.397 (3)
C3—H3	0.9500	C32—H32	0.9500
C4—C5	1.436 (2)	C33—O31	1.344 (2)
C4—H4	0.9500	C33—C34	1.378 (3)
C5—C6	1.395 (2)	C34—C35	1.373 (3)
C6—C7	1.395 (2)	C34—H34	0.9500
C6—C11	1.495 (2)	C35—H35	0.9500
C7—C8	1.440 (2)	O31—C36	1.435 (2)
C8—C9	1.348 (2)	C36—H36A	0.9800
C8—H8	0.9500	C36—H36B	0.9800
C9—C10	1.435 (2)	C36—H36C	0.9800
C9—H9	0.9500	Cl1—Cl1^ii^	0.5162 (19)
C10—C1^i^	1.388 (2)	Cl1—O2^ii^	1.228 (6)
C11—C16	1.392 (2)	Cl1—O4^ii^	1.361 (8)
C11—C12	1.395 (2)	Cl1—O2	1.391 (5)
C12—C13	1.389 (2)	Cl1—O4	1.396 (8)
C12—H12	0.9500	Cl1—O3	1.425 (3)
C13—C14	1.381 (3)	Cl1—O1	1.447 (2)
C13—H13	0.9500	Cl1—O3^ii^	1.931 (3)
C14—C15	1.381 (3)	O1—Cl1^ii^	1.447 (2)
C14—H14	0.9500	O2—O4^ii^	0.524 (14)
C15—C16	1.387 (2)	O2—Cl1^ii^	1.228 (6)
C15—H15	0.9500	O3—Cl1^ii^	1.931 (3)
C16—H16	0.9500	O4—O2^ii^	0.524 (14)
C17—C18	1.381 (3)	O4—Cl1^ii^	1.361 (8)
			
N1^i^—Fe1—N1	180.00 (4)	C19—C18—H18	119.8
N1^i^—Fe1—N2^i^	88.56 (5)	C20—C19—C18	120.21 (19)
N1—Fe1—N2^i^	91.44 (5)	C20—C19—H19	119.9
N1^i^—Fe1—N2	91.44 (5)	C18—C19—H19	119.9
N1—Fe1—N2	88.56 (5)	C21—C20—C19	119.64 (17)
N2^i^—Fe1—N2	180.00 (8)	C21—C20—H20	120.2
N1^i^—Fe1—N31	88.87 (6)	C19—C20—H20	120.2
N1—Fe1—N31	91.13 (5)	C20—C21—C22	120.27 (19)
N2^i^—Fe1—N31	90.36 (6)	C20—C21—H21	119.9
N2—Fe1—N31	89.64 (6)	C22—C21—H21	119.9
N1^i^—Fe1—N31^i^	91.13 (5)	C17—C22—C21	120.52 (18)
N1—Fe1—N31^i^	88.87 (6)	C17—C22—H22	119.7
N2^i^—Fe1—N31^i^	89.64 (6)	C21—C22—H22	119.7
N2—Fe1—N31^i^	90.36 (6)	C35—N31—C31	116.36 (15)
N31—Fe1—N31^i^	180.0	C35—N31—Fe1	120.90 (12)
C2—N1—C5	105.28 (13)	C31—N31—Fe1	122.74 (12)
C2—N1—Fe1	125.95 (11)	N31—C31—C32	123.34 (17)
C5—N1—Fe1	128.63 (11)	N31—C31—H31	118.3
C10—N2—C7	106.01 (13)	C32—C31—H31	118.3
C10—N2—Fe1	125.58 (11)	C31—C32—C33	119.39 (18)
C7—N2—Fe1	128.37 (11)	C31—C32—H32	120.3
C10^i^—C1—C2	124.42 (15)	C33—C32—H32	120.3
C10^i^—C1—C17	117.87 (14)	O31—C33—C34	125.44 (16)
C2—C1—C17	117.71 (14)	O31—C33—C32	116.77 (17)
N1—C2—C1	126.01 (15)	C34—C33—C32	117.78 (17)
N1—C2—C3	110.04 (14)	C35—C34—C33	119.04 (16)
C1—C2—C3	123.95 (15)	C35—C34—H34	120.5
C4—C3—C2	107.67 (15)	C33—C34—H34	120.5
C4—C3—H3	126.2	N31—C35—C34	124.07 (16)
C2—C3—H3	126.2	N31—C35—H35	118.0
C3—C4—C5	106.88 (15)	C34—C35—H35	118.0
C3—C4—H4	126.6	C33—O31—C36	116.89 (15)
C5—C4—H4	126.6	O31—C36—H36A	109.5
N1—C5—C6	125.70 (15)	O31—C36—H36B	109.5
N1—C5—C4	110.12 (14)	H36A—C36—H36B	109.5
C6—C5—C4	124.16 (15)	O31—C36—H36C	109.5
C5—C6—C7	122.52 (15)	H36A—C36—H36C	109.5
C5—C6—C11	119.09 (14)	H36B—C36—H36C	109.5
C7—C6—C11	118.39 (14)	Cl1^ii^—Cl1—O2^ii^	97.2 (6)
N2—C7—C6	126.14 (14)	Cl1^ii^—Cl1—O4^ii^	83.1 (6)
N2—C7—C8	109.61 (14)	O2^ii^—Cl1—O4^ii^	128.9 (2)
C6—C7—C8	124.24 (15)	Cl1^ii^—Cl1—O2	61.2 (6)
C9—C8—C7	107.24 (14)	O2^ii^—Cl1—O2	127.7 (7)
C9—C8—H8	126.4	O4^ii^—Cl1—O2	21.9 (6)
C7—C8—H8	126.4	Cl1^ii^—Cl1—O4	75.4 (6)
C8—C9—C10	107.31 (14)	O2^ii^—Cl1—O4	21.8 (6)
C8—C9—H9	126.3	O4^ii^—Cl1—O4	123.9 (7)
C10—C9—H9	126.3	O2—Cl1—O4	114.0 (4)
N2—C10—C1^i^	126.50 (15)	Cl1^ii^—Cl1—O3	166.6 (5)
N2—C10—C9	109.82 (14)	O2^ii^—Cl1—O3	86.4 (4)
C1^i^—C10—C9	123.66 (15)	O4^ii^—Cl1—O3	84.7 (4)
C16—C11—C12	118.74 (15)	O2—Cl1—O3	106.5 (4)
C16—C11—C6	120.59 (15)	O4—Cl1—O3	107.4 (4)
C12—C11—C6	120.65 (15)	Cl1^ii^—Cl1—O1	79.73 (4)
C13—C12—C11	120.16 (17)	O2^ii^—Cl1—O1	116.1 (5)
C13—C12—H12	119.9	O4^ii^—Cl1—O1	114.1 (4)
C11—C12—H12	119.9	O2—Cl1—O1	106.5 (4)
C14—C13—C12	120.68 (17)	O4—Cl1—O1	112.0 (4)
C14—C13—H13	119.7	O3—Cl1—O1	110.32 (17)
C12—C13—H13	119.7	Cl1^ii^—Cl1—O3^ii^	9.8 (3)
C15—C14—C13	119.41 (16)	O2^ii^—Cl1—O3^ii^	88.3 (4)
C15—C14—H14	120.3	O4^ii^—Cl1—O3^ii^	85.6 (4)
C13—C14—H14	120.3	O2—Cl1—O3^ii^	64.1 (4)
C14—C15—C16	120.43 (17)	O4—Cl1—O3^ii^	66.4 (4)
C14—C15—H15	119.8	O3—Cl1—O3^ii^	162.2 (3)
C16—C15—H15	119.8	O1—Cl1—O3^ii^	87.28 (13)
C15—C16—C11	120.57 (16)	Cl1—O1—Cl1^ii^	20.54 (8)
C15—C16—H16	119.7	O4^ii^—O2—Cl1^ii^	97.4 (13)
C11—C16—H16	119.7	O4^ii^—O2—Cl1	75.8 (13)
C18—C17—C22	118.85 (16)	Cl1^ii^—O2—Cl1	21.61 (13)
C18—C17—C1	120.18 (16)	Cl1—O3—Cl1^ii^	3.54 (12)
C22—C17—C1	120.96 (16)	O2^ii^—O4—Cl1^ii^	82.2 (13)
C17—C18—C19	120.50 (19)	O2^ii^—O4—Cl1	60.7 (12)
C17—C18—H18	119.8	Cl1^ii^—O4—Cl1	21.53 (14)

Symmetry codes: (i) −*x*+1, −*y*+1, −*z*+1; (ii) −*x*+1, *y*, −*z*+1/2.

### Hydrogen-bond geometry (Å, º)

**Table:**

*D*—H···*A*	*D*—H	H···*A*	*D*···*A*	*D*—H···*A*
C35—H35···O4^ii^	0.95	2.55	3.103 (8)	117
C36—H36*C*···O1	0.98	2.64	3.551 (3)	154

Symmetry code: (ii) −*x*+1, *y*, −*z*+1/2.
